# Pancreatic Stellate Cells (PSCs) express Cyclooxygenase-2 (COX-2) and pancreatic cancer stimulates COX-2 in PSCs

**DOI:** 10.1186/1476-4598-4-27

**Published:** 2005-08-05

**Authors:** Seiya Yoshida, Michael Ujiki, Xian-Zhong Ding, Carolyn Pelham, Mark S Talamonti, Richard H Bell, Woody Denham, Thomas E Adrian

**Affiliations:** 1 Department of Surgery and Robert H Lurie Comprehensive Cancer Center, Northwestern University Feinberg School of Medicine, 333 East Superior 4–713, Chicago, IL 60611, USA

## Abstract

**Background:**

Cyclooxygenase 2 (COX-2), the inducible form of prostaglandin G/H synthase, is associated with several human cancers including pancreatic adenocarcinoma. Pancreatic stellate cells (PSCs) play a central role in the intense desmoplasia that surrounds pancreatic adenocarcinoma. The present study examined COX-2 expression in PSCs. PSCs isolated from normal rats, were cultured and exposed to conditioned medium (CM) from the human pancreatic cell line, PANC-1.

**Methods:**

COX-2 expression was evaluated by immunostaining and western blotting. Proliferation of PSCs was determined by thymidine incorporation and cell counting.

**Results:**

COX-2 was found to be constitutively expressed in PSCs, and COX-2 protein was up-regulated by PANC-1 CM. Moreover, the induction of COX-2 by PANC-1 CM was prevented by U0126, an extracellular signal-regulated kinase (ERK) 1/2 inhibitor suggesting that activation of ERK 1/2 is needed for stimulation of COX-2. Finally, NS398, a selective COX-2 inhibitor, reduced the growth of PSCs by PANC-1 CM, indicating that activation of COX-2 is required for cancer stimulated PSC proliferation.

**Conclusion:**

The results suggest that COX-2 may play an important role in the regulation of PSC proliferation in response to pancreatic cancer.

## Background

Vitamin A-containing cells were first reported in 1982 by Watari et al. in vitamin A loaded mice using fluorescence and electron microscopy [1]. This cell type was subsequently identified by electron microscopy in normal rat and human pancreatic tissues [2]. These cells were identified as pancreatic stellate cells (PSCs) by Apte et al and Bachem et al in 1998 [3,4]. In the normal pancreas, stellate cells are quiescent and can be identified by the presence of vitamin A-containing lipid droplets in the cytoplasm. In response to pancreatic injury or inflammation, PSCs are transformed ("activated") from quiescent phenotypes into highly proliferative myofibroblast-like cells which express the cytoskeletal protein α-smooth muscle actin (α-SMA), and produce type I collagen and other extracellular matrix components. Many of the morphological and metabolic changes associated with the activation of PSCs in animal models of fibrosis also occur when these cells are cultured on plastic in serum-containing medium.

Activated PSCs have also been implicated in the deposition of extracellular matrix components in pancreatic adenocarcinoma [5]. In patients with pancreatic cancer, an intense, interstitial, fibrillar staining for PSCs is evident in the peritumoral fibrous regions. Procollagen I staining colocalized with α-SMA to these fibroblast-shaped cells suggests that they are responsible for the deposition of matrix components and the desmoplastic reaction that surrounds the pancreatic tumor [5].

Cyclooxygenases (COXs) are key rate-limiting enzymes involved in the conversion of arachidonic acid to prostaglandin (PG) H2, the precursor of a variety of compounds including PGs, prostacyclin, and thromboxanes. Two isozymes are found in mammalian tissues, COX-1 and COX-2. COX-1 is expressed constitutively in a wide variety of tissues, where it is involved in the maintenance of tissue homeostasis. In contrast, COX-2, which is not expressed in resting cells, is the inducible form of the enzyme responsible for PG production at sites of inflammation. Growth factors, cytokines, tumor promoters, and other inflammatory mediators can induce COX-2 expression [6,7]. COX-2 expression and activity is up-regulated in pancreatic cancer, but absent in normal pancreatic acinar and duct cells [8-10]. Some scattered cells in normal pancreatic tissues express COX-2 [11,12].

The current study revealed that COX-2 is expressed in primary cultured PSC. Furthermore, conditioned media from pancreatic cancer stimulates PSC proliferation and COX-2 expression. The increase in PSC proliferation in response to conditioned media is prevented by inhibition of COX-2.

## Results

### COX-2 in primary cultured PSCs

**Figure 1:**
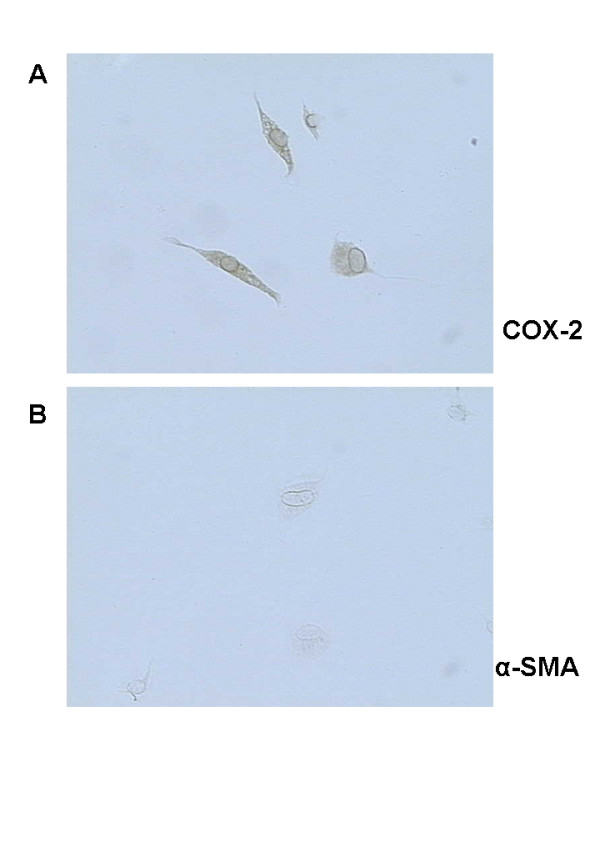
Immunostaining of COX-2 and α-smooth muscle actin (α-SMA) in pancreatic stellate cells (PSCs) after one day in culture. (A) Immunostaining of COX-2 in quiescent PSCs. All PSCs stained for COX-2. (B) Immunostaining of α-SMA in quiescent PSCs. PSCs did not stain for α-SMA. Magnification 400×.

**Figure 2:**
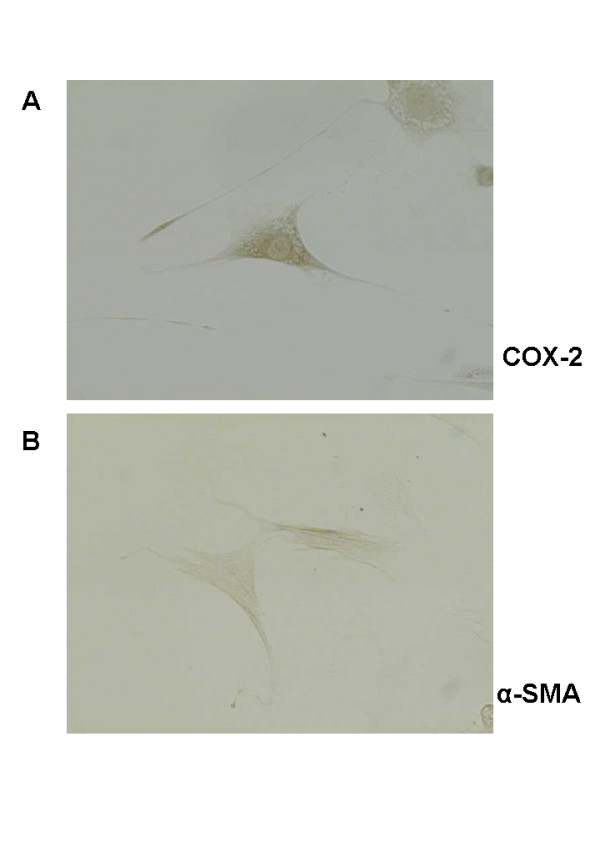
Immunostaining of COX-2 and α-smooth muscle actin (α-SMA) in pancreatic stellate cells (PSCs) after 10 days in culture. (A) Immunostaining of COX-2 in activated PSCs. (B) Immunostaining of α-SMA in activated PSCs. Magnification 400×. All PSCs stained for both COX-2 and α-SMA.

In early primary PSCs, cytoplasmic COX-2 staining was detected (Figure 1). However, early primary cultured PSCs (quiescent cells) were α-SMA negative (Figure 1). After passage, PSCs flattened and developed long cytoplasmic extensions (activated PSCs), and showed positive immunostaining for COX-2 and α-SMA (Figure 2).

### COX-2 protein in culture-activated PSCs

**Figure 3:**
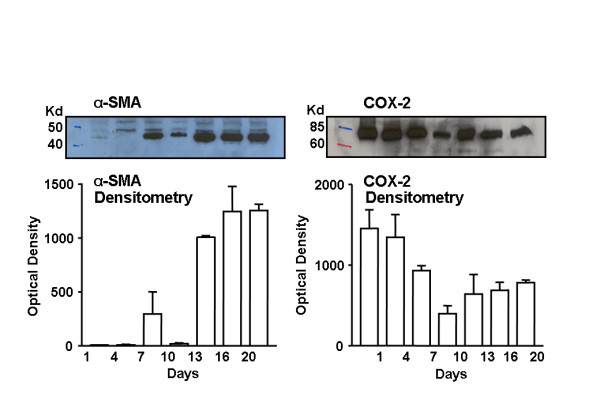
Induction of COX-2 and α-smooth muscle actin (SMA) protein in pancreatic stellate cells (PSCs). After isolation of PSCs, equal amounts of protein from the cell lysates were loaded by SDS-PAGE and immunoblotted with COX-2 or α-SMA antibodies. Upper panels show representative western blots and lower panels show the densitometry data from all experiments. PSCs expressed α-SMA after seven days in culture. In contrast, PSCs expressed COX-2 throughout this time period.

On days one and four in primary culture, PSCs expressed low levels of α-SMA (Figure 3). Between day 7 and day 20, α-SMA expression increased substantially (Figure 3). In. contrast, the COX-2 protein was detected in primary cultured PSC from day 1 through day 20 (Figure 3).

### Expression of COX-2 protein in PSCs was increased by PANC-1 CM

**Figure 4:**
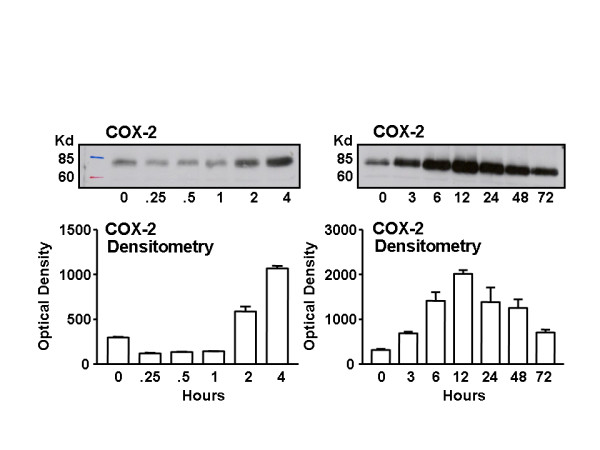
The expression of COX-2 protein in pancreatic stellate cells (PSCs) was increased by cancer conditioned medium (PANC-1 CM). Stellate cells were isolated and cultured in media containing 10% serum for 12 days. Then, following 18-hour culture in 1% serum medium, cells were treated with PANC-1 CM for the indicated times. Upper panels are representative western blots showing the effect of PANC-1 CM on COX-2 expression over two different time-courses. The lower panels show that densitometric analysis western blots from the separate experiments. PANC-1 CM caused a rapid and sustained up-regulation of COX-2 protein, which was maximally increased after 12 hours and remained elevated.

PSCs were treated with PANC-1 CM for 0.5, 1, 3, 6, 12, 24, 48, and 72 hours. PANC-1 CM caused sustained up-regulation of the COX-2 protein, which was maximally increased after 12 hours and remained elevated for at least 24 hours (Figure 4).

### The increase in expression of COX-2 protein in PSCs by PANC-1 CM was inhibited by U0126

**Figure 5:**
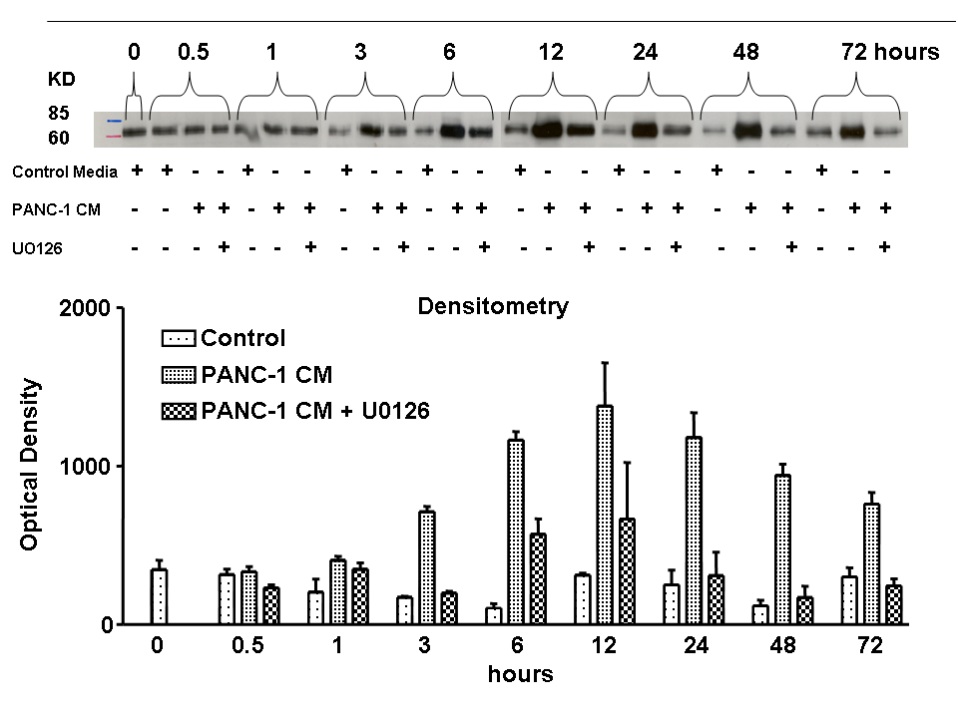
Effects of U0126, a specific inhibitor of ERK activation on cancer conditioned medium (PANC-1 CM)-induced COX-2 expression in pancreatic stellate cells (PSCs) by Western blot. Following 18-hour culture in 1% serum medium, cells were treated with control medium and PANC-1 CM in the absence and presence of 10 μM U0126 for the indicated times. Equal amounts of protein from the cell lysates were loaded by SDS-PAGE and immunoblotted with COX-2 antibody. The upper panel is a representative western blot and the lower panel shows densitometric analysis of the western blots from the separate experiments. The increase in expression of the COX-2 protein in PSCs in response to PANC-1 CM was abolished by U0126. This figure is representative of three separate experiments.

PSCs were treated with PANC-1 CM and control medium and PANC-1 CM with the mitogen-activated protein kinase kinases (MEK) inhibitor U0126 (10 μM) for 0.5, 1, 3, 6, 12, 24, 48, 72 hours. U0126 significantly inhibited PANC-1-induced expression of COX-2 (Figure 5).

### NS398 inhibits cell proliferation of PSCs stimulated by PANC-1 CM

**Figure 6:**
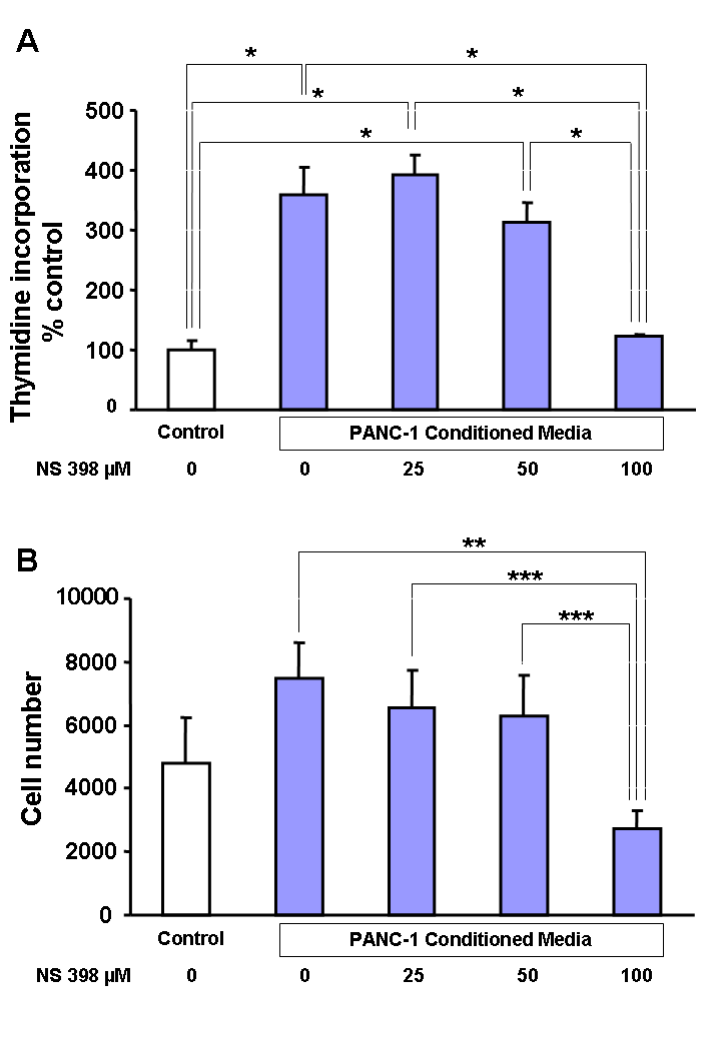
Effect of cancer all conditioned medium (PANC-1 CM) with or without NS398. (A) Inhibition of COX-2 activity with NS398 decreased DNA synthesis (thymidine incorporation) in pancreatic stellate cells (PSCs). Results are expressed as percent of control. (B) NS398 inhibited cell growth in PSCs. Results are expressed as mean ± SEM from three separate experiments. * P < 0.001; ** P < 0.01; *** P < 0.05.

Since COX-2 is increased by PANC-1 CM, the role of COX-2 in PANC-1 CM-induced PSC proliferation was investigated using a specific COX-2 inhibitor, NS398. PANC-1 CM increased PSC thymidine incorporation as well as cell number compared to control medium (Figure 6). Inhibition of COX-2 with NS398 resulted in a concentration-dependent decrease in thymidine incorporation and cell number.

## Discussion

There is accumulating evidence that PSCs play a role in the development of pancreatic fibrosis [13,14]. Little is known regarding the relationship between PSCs and pancreatic cancer, or the role of COX-2.

The present study revealed that PSCs express COX-2 constitutively and when activated. The two isoforms of COX, COX-1 and COX-2, differ in many respects. COX-1 is a housekeeping gene that is expressed in most tissue, while COX-2 is not detected in most normal tissues. In the pancreas, islet cells display a strong expression of COX-2 [9]; however, some scattered basal cells in normal pancreas express COX-2 as well, though less than seen in islet cells [11,12]. In hepatic stellate cells (HSCs) which are similar to PSCs, COX-2 expression is virtually undetectable by Western blot analysis in protein extracts obtained from freshly isolated HSC [15]. However, serum-deprived unstimulated HSC express low levels of the COX-2 protein and expression is dramatically enhanced in response to IL-1α, TNF α or endothelin-1 [15,16]. The present study suggests that COX-2 expression is independent of the activation status in isolated PSCs. While there is no marked expression of COX-2 in desmoplastic areas of pancreatic cancers [8-10], it is possible that the enzyme is up-regulated early in the activation of stellate cells *in vivo *but increased expression may not be required for maintenance of stellate function once activated.

Stimulation of PSC by PANC-1 CM increased the expression of COX-2. Oncogenes, growth factors, cytokines, chemotherapy and tumor promoters stimulate COX-2 transcription via protein kinase C and RAS-mediated signaling. Stimulation of either protein kinase C or RAS-mediated signaling enhances mitogen-activated protein (MAP) kinase activity, which in turn, activates transcription of COX-2 [17]. We have previously reported that PANC-1 CM enhances ERK 1/2 activation and growth of PSCs [18]. We speculate that a growth factor is responsible for these effects, however, our attempts to identify the candidate using receptor antagonists and immunoneutralization have not been successful. Inhibition of ERK1/2 phosphorylation by U0126 prevented the PANC-1 CM-stimulated increase in PSC COX-2 protein production. In previous studies U0126 alone had no effect on ERK1/2 or COX-2 expression [19,20]. This suggests that the MAP kinase pathway plays a role in cancer-induced stimulation of COX-2 in PSCs. The reported biological consequences of COX-2 up-regulation include growth stimulation inhibition of apoptosis [21], increased metastatic potential [22] and promotion of angiogenesis [23]. Increased expression of COX-2 in PSCs by PANC-1 CM may contribute to tumor progression.

Finally, the proliferation of PSCs was inhibited by treatment with NS398, a COX-2 inhibitor. In pancreatic carcinomas, COX-2 is overexpressed and NS398 inhibits tumor growth [8,9,24]. This COX-2 inhibitor alone has no effect on expression of COX-2 or ERK1/2 and shows no toxicity at the concentration used in the present studies [20,24]. Recent studies have demonstrated a role for the COX-2 enzyme and PGE2 in the regulation of epithelial cell growth and angiogenesis [23,25,26]. These properties will need to be studied further in pancreatic adenocarcinoma and stellate cells. NS-398 has been previously shown to inhibit cell proliferation of colorectal carcinoma by inducing apoptosis in a COX-2-independent fashion [27]. More studies are needed to confirm the mechanism of inhibition by NS398.

## Conclusion

The COX-2 protein is up-regulated in pancreatic stellate cells by pancreatic cancer-conditioned media. The induction of COX-2 by pancreatic cancer cells is mediated by extracellular signal-regulated kinases 1/2 (ERK1/2). The COX-2 induction by pancreatic cancer cells is involved in mediating PSC proliferation. Therefore, COX-2 may play an important role in the regulation of desmoplasia in pancreatic cancer and inhibition of this enzyme may prevent or reduce this response.

## Materials and methods

### Materials

Iscove's modified Dulbecco's medium (IMDM), Dulbecco's modified Eagle's medium (DMEM), albumin, and pronase were purchased from Sigma Chemical (St Louis, MO). Fetal bovine serum (FBS), glutamine, and antibiotics were purchased from Mediatech, Inc. (Herndon, VA). Collagenase P was purchased from the Roche Diagnostics Corporation (Indianapolis, IN), and deoxyribonuclease from Amersham Biosciences (Piscataway, NJ). Nycodenz was obtained from Nycomed Pharma AS (Oslo, Norway). U0126, a specific inhibitor of extracellular signal-regulated kinase (ERK) activation, was obtained from Calbiochem (San Diego, CA). NS398, COX-2 inhibitor was obtained from Cayman Chemicals (Ann Arbor, MI). ^3^H-methyl thymidine was purchased from ICN Pharmaceuticals (Costa Mesa, CA).

### Animals

Male Sprague-Dawley rats weighing 200–250 g were used in accordance with standard institutional animal welfare guidelines, and protocols were approved by the Institutional Animal Care and Use Committee, Northwestern University School of Medicine.

### Isolation and culture of PSCs

Rat PSCs were isolated as previously described [3]. Briefly, the pancreas was digested with a mixture of collagenase P and pronase and deoxyribonuclease in Gey's balanced salt solution. The resulting suspension of cells was centrifuged in a 28.7% Nycodenz gradient at 1400 *g *for 23 minutes. Stellate cells then separated into a hazy band just above the interface of the Nycodenz solution and the aqueous buffer. Cells were harvested, washed, and resuspended in IMDM containing 10% FBS, 4 mmol/l glutamine, and antibiotics. PSCs were all used within two passages following isolation.

### Conditioned medium

The poorly differentiated pancreatic adenocarcinoma cell line PANC-1 (American Type Tissue Culture, Rockville, MD) was grown in DMEM in 75 cm^2 ^flasks. When the cells reached confluence, the serum-containing medium was removed and the cells were cultured in 20 ml of serum-free medium. After 24 hours, the medium was collected and the peptide containing fraction obtained by semi-purifying on a Sep-Pak Plus C18 Cartridge (Waters, Milford, MA). After washing, Sep-Paks were eluted with 50% acetonitrile (EM Science, Gibbstown, NJ) with 0.1% trifluoroacetic acid (J. T. Baker, Phillipsburg, NJ). The eluates were lyophilized and reconstituted in fresh IMDM with 1% FBS, forming what we refer to as PANC-1 conditioned medium (CM). In total extracts of 100 ml pf PANC-1 conditioned media were purified and reconstituted in 20 ml of media for PSC culture. Control medium consisted of only serum-free medium without PANC-1 cells which underwent are same Sep-Paking procedure. The eluates were also lyophilized, and then reconstituted in fresh IMDM with 1% FBS.

### Immunostaining

COX-2 and α-SMA expression in PSCs was evaluated by immunohistochemical staining. Cultured PSCs were grown directly on glass coverslips in six-well plates, and immunostained for COX-2 using peroxidase-labeled streptavidin for immunohistochemistry (KPL, Inc., Maryland) according to the manufacturer's instructions. Cells were fixed for 30 minutes in acetone at -20°C. Thereafter, glass coverslips were air-dried and stored at 4°C until the cells were stained. Endogenous peroxidase activity was blocked by incubation in methanol with 0.3% hydrogen peroxidase for 30 minutes. After immersion in normal goat serum for 30 minutes, the slides were incubated with COX-2 (murine) polyclonal antibody (Cell Signaling Technology, Inc., Beverly, MA) diluted 1:200 in tris-buffered saline (TBS) 1× bovine serum albumin and stored in a humid chamber overnight at 4°C. The slides were incubated with anti-mouse immunoglobulins for 10 minutes at 37°C, followed by peroxidase conjugated streptavidin for 30 minutes at room temperature. Finally, color was developed incubating the slides for 8 minutes, with diaminobenzine (DAB Reagent Set; KPL, Inc., Maryland). Expression of α-SMA was examined in a similar manner by using monoclonal anti-α-SMA antibody (Sigma, St Louis, MI).

### Protein extraction

Protein concentrations in the cell lysates were measured by the method of Lowry et al. [28] using bovine serum albumin as the standard.

### ^3^H-methyl thymidine incorporation

Following the treatment of cells with PANC-1 CM for 48 hours, DNA synthesis was measured by adding to each well 0.5 μCi of ^3^H-methyl thymidine and incubating these plates for the final 24 hours. The cell protein was precipitated with 10% trichloroacetic acid overnight, washed twice with phosphate buffered saline (PBS) and then dissolved by adding 0.25 ml of 0.5 mol/l NAOH to each well. Incorporation of ^3^H-methyl thymidine into DNA was measured by adding 1 ml of scintillation cocktail (ScintiSafe Plus 50%, S × 25-5, Fischer Scientific, Pittsburgh, PA) followed by count measurements using the Wallac WinSpectral liquid scintillation counter (Wallac Turku, Finland).

### Cell counts

Cells were washed with PBS, harvested by trypsinization using 0.5% trypsin-0.2% EDTA, resuspended in 100 μl culture medium, and counted using the Guava Personal Cytometer (Guava Technologies, Inc., Burlingame, CA).

### Western blotting

Expression of COX-2 and α-SMA were detected by Western blotting. Protein extracts (5 μg) from each sample were separated by gel electrophoresis using a 10% sodium dodecyl sulfate-polyacrylamide gel (SDS-PAGE). Known molecular weight protein standards were run alongside the samples. Separated proteins were then transferred to a nitrocellulose membrane (Bio-Rad, Hercules, CA), which was incubated for one hour at room temperature in blocking buffer (TBS and 0.1% Tween 20 with 5% nonfat dry milk). A murine COX-2 polyclonal antibody was diluted 1:2000 buffer (TBS and 0.1% Tween 20 with 5% nonfat dry milk). After incubation with the primary antibody overnight at 4°C, the membrane was exposed to the secondary antibody with gentle agitation for one hour at room temperature. Western Blots were visualized using Chemiluminescence Luminol Reagent (Santa Cruz Biotechnology, Inc., CA). α-SMA expression was examined in a similar manner by using monoclonal anti-α-SMA antibodies. Bands from individual western blots were quantified densitometrically and the mean ± SEM for each time point or concentration calculated for presentation.

### Statistical analysis

All experiments were repeated at least twice. Data are expressed as mean ± SEM. Statistical analysis was performed using ANOVA with the Prism software package (GraphPad, San Diego, CA).

## Authors' contributions

SY, MU, CP and WD participated in the PSC isolation and western blot experiments, cell culture experiments and drafted the manuscript. TA, XD, RB, MT and WD participated in the design of the study and trouble-shooting of the experiments. All authors read and approved the final manuscript.

## References

[B1] Watari N, Hotta Y, Mabuchi Y (1982). Morphological studies on a vitamin A-storing cell and its complex with macrophage observed in mouse pancreatic tissues following excess vitamin A administration. Okajimas Folia Anat Jpn.

[B2] Ikejiri N (1990). The vitamin-A storing cells in the human and rat pancreas. Kurume Med J.

[B3] Apte MV, Haber PS, Applegate TL, Norton ID, McCaughan GW, Korsten MA, Pirola RC, Wilson JS (1998). Periacinar stellate shaped cells in rat pancreas: identification, isolation, and culture. Gut.

[B4] Bachem MG, Schneider E, Gross H, Weidenbach H, Schmid RM, Menke A, Siech M, Beger H, Grunert A, Adler G (1998). Identification, culture, and characterization of pancreatic stellate cells in rats and humans. Gastroenterology.

[B5] Yen TW, Aardal NP, Bronner NP, Thorning DR, Savard CE, Lee SP, Bell RH (2002). Myofibroblasts are responsible for the desmoplastic reaction surrounding human pancreatic carcinomas. Surgery.

[B6] Ristimäki A, Garfinkel S, Wessendorf J, Maciag T, Hla T (1994). Induction of cyclooxygenase-2 by interleukin-1α; evidence for post-transcriptional regulation. J Biol Chem.

[B7] Smith WL, DeWitt DL, Garavito RM (2000). Cyclooxygenases: structutal, cellular, and molecular biology. Annu Rev Biochem.

[B8] Molina MA, Sitja-Arnau M, Lemoine MG, Frazier M, Sinicrope FA (1999). Increased cyclooxygenase-2 expression in human pancreatic carcinomas and cell lines: growth inhibition by nonsteroidal anti-inflammatory drugs. Cancer Res.

[B9] Okami J, Yamamoto H, Fujiwara Y, Tsujie M, Kondo K, Noura S, Oshima S, Nagano H, Dono K, Umeshita K, Ishikawa O, Sakon M, Matsuura N, Nakamori S, Monden M (1999). Overexpression of cyclooxygenase-2 in carcinoma of the pancreas. Clin Cancer Res.

[B10] Tucker ON, Dannenberg AJ, Yang EK, Zhang F, Teng L, Daly JM, Soslow RA, Masferrer JL, Woerner BM, Koki AT, Fahey TJ (1999). Cyclooxygenase-2 expression is up-regulated in human pancreatic cancer. Cancer Res.

[B11] Merati K, Siadaty MS, Andea A, Sarkar F, Ben-Josef E, Mohammad R, Philip P, Shields AF, Vaitkevicius V, Grignon DJ, Adsay NV (2001). Expression of inflammatory modulator COX-2 in pancreatic ductal adenocarcinoma and its relationship to pathologic and clinical parameters. Am J Clin Oncol.

[B12] Maitra A, Ashfaq R, Gunn CR, Rahman A, Yeo CJ, Sohn TA, Cameron JL, Hruban RH, Wilentz RE (2002). Cyclooxygenase 2 expression in pancreatic adenocarcinoma and pancreatic intraepithelial neoplasia: an immunohistochemical analysis with automated cellular imaging. Am J Clin Pathol.

[B13] Wells RG, Crawford JM (1998). Pancreatic stellate cells: the new stars of chronic pancreatitis?. Gastroenterology.

[B14] Haber PS, Keogh GW, Apte MV, Moran CS, Stewart NL, Crawford DH, Pirola RC, McCaughan GW, Ramm GA, Wilson JS (1999). Activation of pancreatic stellate cells in human and experimental pancreatic fibrosis. Am J Pathol.

[B15] Efsen E, Bonacchi A, Pastacaldi S, Valente AJ, Wenzel UO, Tosti-Guerra C, Pinzani M, Laffi G, Abboud HE, Gentilini P, Marra F (2001). Agonist-specific regulation of monocyte chemoattractant protein-1 expression by cyclooxygenase metabolites in hepatic stellate cells. Hepatology.

[B16] Gallois C, Habib A, Tao J, Moulin S, Maclouf J, Mallat A, Lotersztajn S (1998). Role of NF-κB in the antiproliferative effect of endothelin-1 and tumor necrosis factor-α in human hepatic stellate cells. Involvement of cyclooxygenase-2. J Biol Chem.

[B17] Subbaramaiah K, Dannenberg AJ (2003). Cyclooxygenase 2: a molecular target for cancer prevention and treatment. Trends Pharmacol Sci.

[B18] Yoshida S, Yokota T, Ujiki M, Ding XZ, Pelham C, Adrian TE, Talamonti MS, Bell RH, Denham W (2004). Pancreatic cancer stimulates pancreatic stellate cell proliferation and TIMP-1 production through the MAP kinase pathway. Biochem Biophys Res Commun.

[B19] Ding XZ, Tong WG, Adrian TE (2003). Multiple Signal Pathways Are Involved in the Mitogenic Effect of 5(S)-HETE in Human Pancreatic Cancer. Oncology.

[B20] Gao J, Niwa K, Takemura M, Sun W, Onogi K, Wu Y, Seishima M, Mori H, Tamaya T (2005). Significant anti-proliferation of human endometrial cancer cells by combined treatment with a selective COX-2 inhibitor NS398 and specific MEK inhibitor U0126. Int J Oncol.

[B21] Tsujii M, DuBois RN (1995). Alterations in cellular adhesion and apoptosis in epithelial cells overexpressing prostaglandin endoperoxide synthase 2. Cell.

[B22] Tsujii M, Kawano S, DuBois RN (1997). Cyclooxygenase-2 expression in human colon cancer cells increases metastatic potential. Proc Natl Acad Sci USA.

[B23] Tsujii M, Kawano S, Tsuji S, Sawaoka H, Hori M, Dubois RN (1998). Cyclooxygenase regulates angiogenesis induced by colon cancer cells. Cell.

[B24] Ding XZ, Tong WG, Adrian TE (2000). Blockade of cyclooxygenase-2 inhibits proliferation and induces apoptosis in human pancreatic cancer cells. Anticancer Res.

[B25] Leahy KM, Ornberg RL, Wang Y, Zweifel BS, Koki AT, Masferrer JL (2002). Cyclooxygenase-2 inhibition by celecoxib reduces proliferation and induces apoptosis in angiogenic endothelial cells in vivo. Cancer Res.

[B26] Jabbour HN, Boddy SC (2003). Prostaglandin E_2 _induces proliferation of glandular epithelial cells of the human endometrium via extracellular regulated kinase 1/2-mediated pathway. J Clin Endocrinol Metab.

[B27] Elder DJ, Halton DE, Hague A, Paraskeva C (1997). Induction of apoptotic cell death in human colorectal carcinoma cell lines by a cyclooxygenase-2 (COX-2)-selective nonsteroidal anti-inflammatory drug: independence from COX-2 protein expression. Clin Cancer Res.

[B28] Lowry OH, Rosebrough NJ, Farr AL, Randall RJ (1951). Protein measurement with the folin phenol reagent. J Biol Chem.

